# Maternal Antibiotic-Induced Early Changes in Microbial Colonization Selectively Modulate Colonic Permeability and Inducible Heat Shock Proteins, and Digesta Concentrations of Alkaline Phosphatase and TLR-Stimulants in Swine Offspring

**DOI:** 10.1371/journal.pone.0118092

**Published:** 2015-02-17

**Authors:** Marie-Edith Arnal, Jing Zhang, Clett Erridge, Hauke Smidt, Jean-Paul Lallès

**Affiliations:** 1 Food and Digestive, Central and Behavioral Adaptation Department, French National Institute for Research in Agriculture, Saint-Gilles, France; 2 Laboratory of Microbiology, Wageningen University, Wageningen, The Netherlands; 3 Department of Cardiovascular Sciences, Glenfield General Hospital, University of Leicester, Leicester, United Kingdom; Charité, Campus Benjamin Franklin, GERMANY

## Abstract

Elevated intake of high energy diets is a risk factor for the development of metabolic diseases and obesity. High fat diets cause alterations in colonic microbiota composition and increase gut permeability to bacterial lipopolysaccharide, and subsequent low-grade chronic inflammation in mice. Chronic inflammatory bowel diseases are increasing worldwide and may involve alterations in microbiota-host dialog. Metabolic disorders appearing in later life are also suspected to reflect changes in early programming. However, how the latter affects the colon remains poorly studied. Here, we hypothesized that various components of colonic physiology, including permeability, ion exchange and protective inducible heat shock proteins (HSP) are influenced in the short- and long-terms by early disturbances in microbial colonization. The hypothesis was tested in a swine model. Offspring were born to control mothers (n = 12) or mothers treated with the antibiotic (ATB) amoxicillin around parturition (n = 11). Offspring were slaughtered between 14 and 42 days of age to study short-term effects. For long-term effects, young adult offspring from the same litters consumed a normal or a palm oil-enriched diet for 4 weeks between 140 and 169 days of age. ATB treatment transiently modified maternal fecal microbiota although the minor differences observed for offspring colonic microbiota were nonsignificant. In the short-term, consistently higher HSP27 and HSP70 levels and transiently increased horseradish peroxidase permeability in ATB offspring colon were observed. Importantly, long-term consequences included reduced colonic horseradish peroxidase permeability, and increased colonic digesta alkaline phosphatase (AP) and TLR2- and TLR4-stimulant concentrations in rectal digesta in adult ATB offspring. Inducible HSP27 and HSP70 did not change. Interactions between early ATB treatment and later diet were noted for paracellular permeability and concentrations of colonic digesta AP. In conclusion, our data suggest that early ATB-induced changes in bacterial colonization modulate important aspects of colonic physiology in the short- and long-terms.

## Introduction

Inflammatory bowel diseases (IBD), including Crohn’s disease and ulcerative colitis are chronic gut pathologies afflicting humans at different stages of their life [1]. The ethio-pathogenesis of these diseases is considered multifactorial, with influences of heredity, environment, and even the gut microbiota whose community structure is often perturbed [1]. Major gut functions such as permeability and ion transport are altered in IBD [2,3]. Conversely, colonic chloride and fluid secretion modulate bacterial-epithelial interaction for preventing bacterial translocation [4]. Besides, metabolic diseases, including insulin resistance, type 2-diabetes, obesity, hypertension and cardiovascular diseases causally involve alterations in colonic barrier function and leakage of so-called microbial-associated microbial patterns (MAMPs) like lipopolysaccharide (LPS) into the body, thus leading to chronic low-grade inflammation and disease development [5]. More widely, innate pathogen receptors and their ligands such as LPS are now recognized as linking diet, gut microbiota, inflammation, host metabolic responses and obesity [6,7].

The gut microbiota favors host anatomical, physiological and metabolic development [8]. For example, neonatal bacterial colonization determines gut angiogenesis, epithelial cell renewal, gut permeability and colonic fermentation processes in the colon [8]. Colonic protective HSPs (HSP27 and HSP70) are induced by the commensal microbiota, bacterial components (e.g. LPS; flagellin) and specific fermentation products (e.g. butyrate) [9,10,11,12,13]. The microbiota-host interplay leading to gut HSP expression involves MyD88- and Toll-Like Receptor (TLR)-mediated mechanisms [11,14]. Gut-borne circulating microbial TLR-stimulants like LPS are also suspected to cause metabolic disorders and obesity through triggering inflammation [5,6]. On the other hand, gut chemical-induced inflammation or chronic consumption of very high fat diets promotes changes in colonic microbiota associated with increased luminal concentrations of TLR-stimulants (e.g. LPS) in mice [15,16]. However, little is known on gut TLR-stimulant concentrations in more physiological situations, especially in non-rodent species like pigs.

Early programming of metabolic diseases seems to affect various tissues and organs but very few data have been published on colonic programming and long-term outcomes thus far [17]. A study in rats with intra-uterine growth retardation reported postnatal colonic barrier alteration and longer term alterations in mucin gene expression [18]. Early alterations in colonic transcriptome as consequences of antibiotic treatment of gestating mothers or offspring were reported [19], but functional data and late outcomes are lacking.

In the present work, our hypothesis was that early alterations in neonatal gut microbiota impact colonic functions, including permeability, ion transport and protective HSPs later in life. It was tested in offspring born to sows receiving amoxicillin orally around parturition, and whose colon was investigated between 14 and 42 days of age (short-term study, ST), or at 169 days of age, after offspring had been fed either a normal, low fat (LF) or a high fat (HF), palm oil-containing diet for 28 days (long-term study, LT). Our first report provided insights into small intestinal function [20]. The main outcome of the present report is that specific colonic functional parameters (permeability, luminal TLR-stimulants) were influenced in young and adult offspring born to antibiotic-treated mothers, sometimes in interaction with offspring late diet composition.

## Materials and Methods

### Experimental procedure

Ethics statement: The experiment was designed and executed in 2010 in compliance with French and European law (Decree No. 2001–464 29/05/01, 86/609/CEE) for the care and use of laboratory animals. At that time (2010) getting approval from an ethic committee was not mandatory. One of us (JPL) held the authorization certificate No. 006708 for experimentation on living animals delivered by the French Veterinary Services. INRA Saint-Gilles, including the on-site slaughterhouse has an institutional license (agreement No. A35–622) from the French Veterinary Services.

Data relating to the present publication will be made available upon request.

The experiment has been presented in detail in our previous report [20]. Briefly, 23 sows were used in two successive batches in time, taking into account parity and resistance of selected fecal bacteria to amoxicillin. For each batch, the sows with amoxicillin-sensitive fecal bacteria were assigned to the antibiotic (ATB, n = 11) group in priority, the remaining sows being assigned to the control (CTL, n = 12) group. Groups of sows were located into different rooms of the same farrowing unit, and specific measures were taken to minimize cross contaminations between rooms. Amoxicillin (40 mg/kg body weight, BW) was given orally to the sows with the morning meal (2 kg/day), from 10 days before the estimated farrowing date till 21 days after farrowing. Parturition was not induced.

Litter size in each group was adjusted (n = 12) after birth and male offspring were not castrated later on. Offspring were slaughtered at 14, 21 (for microbiota investigations only), 28 and 42 days after birth (1 per litter, ST study). Weaning occurred at 28 days when offspring were moved to the post-weaning and the fattening units. Homogeneous pairs of males or females within litters were randomly fed either low fat (LF) or HF diet between 140 and 169 days of age (n = 10 litters per treatment) (LT study). The fat source used to prepare the HF diet was palm oil. Chronic HF diet consumption is a stress generating gut barrier alterations, LPS entry and low grade, metabolic inflammation [5]. A late dietary treatment with contrasted fat contents was used because it allowed us to demonstrate long-term consequences of early events on the small intestine of pigs [20] and rats [21]. The composition of diets given to sows and offspring is available in our previous report (S1 Table, [20]).

**Offspring slaughter and collection of colonic digesta and tissue samples**

Offspring were killed on site in our slaughterhouse by electronarcosis and exsanguination [20]. After laparotomy, 20-cm segments of proximal colon situated 10 cm (ST study) to 30 cm (LT study) after the cecum were collected. Colonic digesta were collected (at d14, d21, d28, d42 and d169) for microbiota analysis and frozen at -20°C. Luminal contents from the cecum and rectum were also collected for determination of alkaline phosphatase (AP) activity concentrations (at d14, d28 and d42, ST study), and AP and TLR-stimulant concentrations (d169, LT study), respectively. Empty colonic segments were then flushed with sterile cold saline. Cross-sectional colonic tissue samples were prepared as follows: 10 cm for immediate use in Ussing chambers for electrophysiology and permeability measurements; 5 cm for immediate fixation in buffered formalin 10%, paraffin embedding and histology of colonic crypts; and two portions of whole tissue of 1-cm (cut in 3–4 pieces) each snap-frozen in liquid nitrogen and then stored at -80°C for HSP and heat shock factor-1 (HSF1) analysis and at -20°C for determination of AP activity concentration in colonic tissue.

**Microbiota analysis**

Microbial composition analysis was carried out in the feces of five sows and in colonic contents of their offspring randomly taken per treatment in the first batch of pigs [20]. Samples were analyzed using the Porcine Intestinal Tract Chip (PITChip) at each slaughter age [22,23]. In brief, the PITChip is an oligonucleotide microarray targeting the 16S ribosomal RNA genes of 627 porcine intestinal microbial species-level phylotypes, grouped into 144 genus-like groups [22,23]. Data were processed first using Agilent’s Feature Extraction Software version 9.1 and second in R (library ‘microbiome’ available from: http://microbiome.github.com) as described previously [20].

**Colonic crypt morphometry**

Colonic tissue sections were prepared using standard procedures and full-size crypt morphometry (depth, width, perimeter and surface area; 10–15 crypts per section) was determined by light microscopy as reported previously [21,24]. An average value for each parameter was calculated per animal prior to statistical analysis.

**Colonic permeability and electrophysiology in Ussing chambers**

At each time-point of interest, pigs (two by two: one CTL and one ATB) were randomly slaughtered at 15–20 min intervals each time, and 3 Ussing chambers were used for investigation of colonic permeability and electrophysiology in each pig. Colonic mucosa was stripped off the muscular layers, cut in 3 contiguous pieces and immediately mounted in lucite chambers (1.13 cm² tissue in contact with buffers) equipped with large glassware buffer containers (8 mL of buffer each side), as described previously [25]. Two chambers were used for measuring colonic electrophysiology parameters in basal conditions (values averaged per tissue), one of these chambers also serving for permeability determination. The third chamber was used for electrophysiology and permeability measurements under oxidative stress using monochloramine (final concentration 1 mM, both sides of the tissue) [26]. The Ringer-bicarbonate buffer contained 16 mmol/L of glucose and 16 mmol/L of mannitol on the serosal and mucosal sides, respectively. Chambers were kept at 39°C and buffers oxygenated all the time. Tissue viability was checked every 30 min by recording tissue electrical potential difference (PD) automatically [25].

Before the start of electrophysiology studies, colonic mucosae were left equilibrating for 20 min before short-circuit current (Isc) and the transepithelial electrical resistance (TEER) were determined under clamp condition (3 mV for 300 ms every 30 s) using Ohm’s law [25]. Colonic mucosa sodium-dependent glucose absorption capacity and chloride secretion capacity were determined after addition of D-glucose (16 mM, mucosal side) and the cholinergic agonist carbachol (10^–3^ M, serosal side), respectively [25]. These measurements were determined in both basal and oxidative condition as explained above, and the latter data were expressed as percentage of the former ones.

Colonic permeability to FITC-dextran 4000 (FD4, MW 4 kDa; Sigma-Aldrich, Saint-Quentin Fallavier, France) and horseradish peroxidase (HRP type II, MW 40 kDa; Sigma-Aldrich were assessed as described previously [27]. FD4 and HRP are considered as markers of para-cellular and trans-cellular permeabilities, respectively, although para-cellular leakage of HRP may also happen under carbachol stimulation [28]. HRP After tissue electrophysiology stabilization as indicated above, both marker probes were added on the mucosal side of Ussing chambers and then serosal fluid (500 μL, immediately replaced by 500 μL of Ringer-glucose) was collected every 30 min for 120 min for marker passage analysis. Fluid FD4 and HRP concentrations were determined by fluorimetry and spectrophotometry, and marker transmucosal flows calculated as detailed previously [27].

**Inducible heat shock proteins and heat shock factor-1**

Frozen colonic tissue was ground in liquid nitrogen and then soluble protein were extracted in borate buffer and protease inhibitor cocktail [29]. Protein concentration was determined using Pierce^TM^ BCA protein assay kit (ref. 23225; Thermo Scientific, Rockford, IL). Tissue concentrations of HSPs (HSP27, HSP70; relative to β-actin as the reference protein) were assayed by Western blotting as previously reported [29]. However, we used for this study new equipment (including: Mini Protean Tetra Cell system; electrophoresis system, Trans Blot Turbo Transfer Starter System) and reagents (including: Tris/Glycine/SDS buffer, pre-casted 12% TGX gels, PVDF membranes and molecular weight standards) for Western blotting, all purchased from Bio-Rad (Marne-La-Coquette, France), as described previously [20]. Western blotting of HSF1, the main transcription factor for HSPs was also conducted as in our previous study [20]. All the technical conditions (incubation times and references of reagents, including primary and secondary antibodies) applied here were the same as previously [20]. Briefly, 10 μg of sample protein were deposited in each well and the electrophoresis was conducted in Tris/Glycine/SDS buffer. After protein transfer, membranes were then blocked in defatted milk powder prepared in Tris buffer saline and 0.1% Tween 20. The membranes, after being cut horizontally at the right location were incubated with anti-HSP or anti-HSF1 antibodies for one part, and with anti- β-actin antibodies for the other part of the membrane. Membranes were washed in TBS Tween buffer before incubation with the second antibodies coupled to horseradish peroxidase. After final membrane washing, protein bands were stained by chemiluminescence and analyzed by imaging for density determination. Results of a given HSP or HSF1 band were expressed as a ratio to β-actin band density as determined for the same sample on the same membrane.

**Alkaline phosphatase activity concentrations in colonic tissue and digesta**

The concentration of bioactive AP (E.C. 3.1.3.1) was determined in colonic tissue homogenates and in digesta as previously reported [21,24]. Colonic AP activity concentrations were expressed per mg of soluble protein, and per g of tissue or g of fresh digesta, depending on the type of samples.

**Determination of TLR-stimulants in rectal contents (LT study)**

The concentrations of TLR-stimulants in feed and rectal contents (LT study) were determined using biocellular quantitative assays specific for TLR2- and TLR4-stimulants, as described elsewhere [15]. Briefly, samples were extracted in phosphate buffer saline and then centrifuged at 13,000 g for 20 min. These extracts were made sterile by filtration (Millipore filters, 0.25 μm, Acrodisc ref. 4612) before being loaded in triplicates on TLR-deficient HEK-293 cell lines. These cells were transfected 3 days before with a firefly luciferase-reporter construct driven by a NF-kB dependent E-selectin promoter, a transfection efficiency control renilla based reporter and constructs coding for either TLR2 or TLR4. Synthetic bacterial lipopeptide (Pam3CSK4) and *Escherichia coli* LPS, as TLR2- and TLR4- stimulants respectively, were used for making standard curves [15].

### Statistical analysis

In order to relate changes in total bacterial community composition to treatment and sampling time (ST) or diet composition (LT), redundancy analysis (RDA) and principal response curves (PRC) testing were performed as described in detail previously, using the CANOCO 4.5 software package (Biometris, Wageningen, the Netherlands) [20]. In brief, PRC analysis is a specific type of RDA [30]. PRC analysis, as used in the present study is able to explain the variation in microbial composition between different experimental groups by including ATB treatment as explanatory variable, and the interaction between treatment and the sampling times or late diet as covariables. The signal intensities for 144 genus-level phylogenetic groups targeted by the PITChip were used as response variables. Significance of the effect of ATB treatment on the variation in microbial composition was tested by Monte Carlo permutation analysis.

Statistical analysis of electrophysiology, permeability and biology data was carried out using the Statistical Analyzing System (SAS). Offspring data were analyzed using SAS MIXED models for testing the effects of treatment (error between litters) and time of slaughter (error within litters) (ST study), and the effects of treatment (between litters) and diet (within litters) (LT study). The models also included the interaction term between early treatment and age of slaughter (ST study) or late diet (LT study). Results are presented as SAS-calculated least-square means (LSmeans) and pooled SEM. LSmeans comparisons for each combination of treatment and time (ST study) or diet (LT study) were made using Bonferroni *post-hoc* comparisons when a tendency (P ≤ 0.10) for an interaction between these terms was observed. Effects were considered significant at P ≤ 0.05 (trends at P ≤ 0.10). Age effects *per se* are not commented because it was not the scope of this study.

## Results

### Zootechnical results

ATB treatment of sows did not influence their reproduction performance, offspring’s growth performance along ST and LT trials, nor dietary energy intake, (LT study) as reported previously [20].

### Short-term experiment

**Microbiota composition in sows and their offspring**

**Fig 1:**
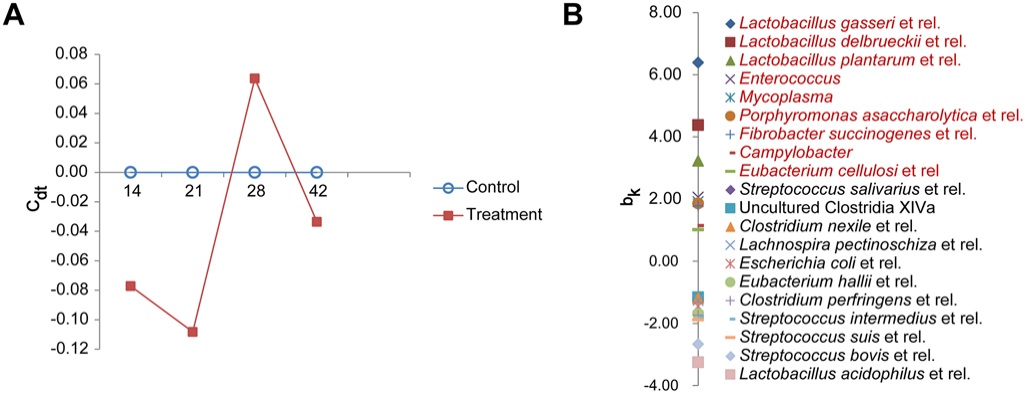
Effects of maternal antibiotic (ATB) treatment on colonic microbiota of offspring between 14 and 42 days of age (weaning at day 28). (n = 5 per treatment). A. Principle Response Curve (PRC) showed no overall significant effects of maternal ATB treatment on colonic microbiota composition in offspring, however, the microbial composition of the ATB group showed a remarkable shift between day 21 and 28 especially for those microbial groups depicted in the B-panel. C_dt_, canonical coefficient. B. The relative contribution of bacteria (red) with a positive species weight (bk) was lower in the ATB group compared to the treatment group on day 14 and 21, but increased between day 21 and 28, and decreased again after weaning at day 28; while those (black) with a negative species weight (bk) showed an opposite trend.

In our previous publication on this extensive experiment [20], we reported that ATB treatment of sows peripartum influenced the composition of their fecal microbiota and ileal microbiota composition of their offspring [20]. Here, in contrast to these earlier observations, PRC analysis showed no significant effects of maternal antibiotic treatment on colonic microbiota composition of offspring during the ST experiment. However, amplitude and direction of minor differences observed between both groups was altered with time (Fig. 1A). Compared to the control, the relative contribution of bacteria (red) with a positive species weight (bk value) in the treated group was lower from day 14 to 21, but increased between day 21 and 28, and decreased again after day 28. Importantly, these bacteria included various species of lactobacilli. The relative contribution of bacteria (black) with a negative species weight showed an opposite trend (Fig. 1B). These bacteria included various species of clostridiae and streptococci.

**Colonic crypt architecture**

The architecture of colonic crypts in the offspring was not significantly influenced by ATB treatment of the mothers, and treatment by age interaction was not significant (S1 Table).

**Colonic permeability and electrophysiology (Ussing chambers)**

Offspring colonic permeability to FD4 in basal or oxidative condition was not influenced by ATB treatment of mothers and the treatment by age was not significant (Table 1). This held true for permeability to HRP in basal condition. However, under oxidative condition, the treatment by age interaction tended towards significance (P = 0.066), showing that HRP permeability tended to be transiently higher in offspring born to ATB-treated mothers at day 14 compared to day 42 (P < 0.10), while these differences were not significant in CTL offspring and were not different from values in ATB offspring aged 42 days (Table 1).

**Table 1:** Mucosal-to-serosal fluxes of FD4 and HRP for basal and monochloramine-stimulated colonic mucosa of pigs born to control or antibiotic-treated sows and slaughtered at different ages (LSmeans and SEM, n = 6–8 per treatment and age).

*Sow’s treatment*	**Control**			**Antibiotics**				Statistics (P =) ^1^		
*Offspring’s age*	**d14**	**d28**	**d42**	**d14**	**d28**	**d42**	**SEM**	**treat**.	**age**	treat.*age
**FD4**										
FD4, basal (ng/cm²/h)	304	213	148	240	235	108	47	0.50	0.012	0.74
FD4, + monochloramine (% basal)	155	172	134	131	225	185	40	0.45	0.51	0.51
**HRP**										
HRP, basal (ng/cm²/h)	19	19	10	32	9	18	10	0.67	0.37	0.53
HRP, + monochloramine (% basal)	191^β^	nd^2^	386^β^	1496^α^	nd	293^β^	444	0.103	0.18	0.066

^1^Treat.: Treatment of sows pre- and post-partum (control *versus* antibiotic); age (d14 and d28, unweaned; d42 weaned from d28); treat*age: treatment by age interaction.

^2^nd: not calculated by the analysis of variance (MIXED model) due to low and unbalanced numbers (n = 2–3) of available HRP data at day 28.

^α, β^ LS means with different superscript letters in a row differ (P<0.10).

Offspring colonic electrophysiology parameters were not influenced by maternal ATB treatment, except for glucose-induced change in Isc in basal condition that tended (P = 0.059) to be higher in offspring born to ATB mothers than in CTL (S2 Table). Interactions were never significant.

**Colonic heat shock proteins and heat shock factor-1**

**Fig 2:**
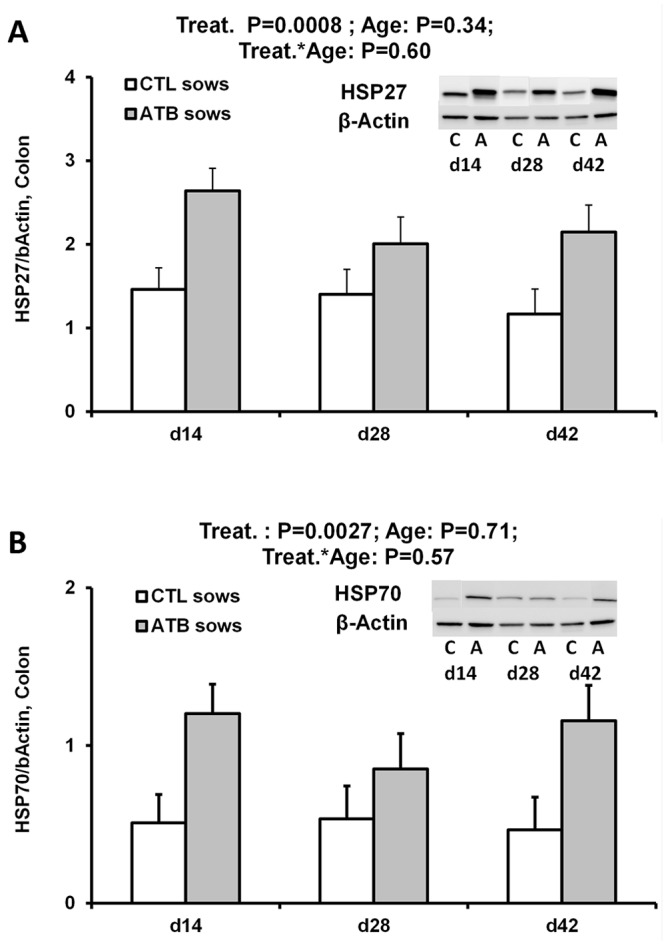
Effects of maternal antibiotic (ATB) treatment on protein expression of inducible heat shock proteins (relative to β-actin) in colonic tissues of offspring between 14 and 42 days of age (weaning at day 28). (LSmeans and SEM, n = 10–12 per treatment and age). A. HSP27. Maternal ATB treatment significantly increased offspring colonic HSP27 levels between d14 and d42 of age (P < 0.001). B. HSP70. Maternal ATB treatment significantly increased offspring colonic HSP70 levels between d14 and d42 of age (P < 0.01). Western blot bands from representative pigs are shown for HSP27 and β-actin (A) and for HSP70 and β-actin (B).

ATB treatment of mothers strongly increased protein concentrations of HSP27 and HSP70 (relative to β-actin) in the colon of their offspring (P < 0.001 and P < 0.01, respectively) (Fig. 2A and 2B). However, offspring colonic HSF1 levels were not influenced by ATB treatment, and treatment by age interaction was not significant [HSF1/β-actin values at 14, 28 and 42 days of age in CTL and ATB offspring: 0.36–0.28–0.24 and 0.38–0.26–0.33 (SEM 0.05), respectively; Treatment, P = 0.47; Age, P = 0.14; Interaction, P = 0.52].

**AP activity concentration in colonic tissues and in cecal and rectal digesta contents**

AP activity concentrations in colonic tissues and in cecal and rectal contents were not influenced by maternal ATB treatment and treatment by age interaction was never significant (S3 Table).

### Long-term experiment

**Microbiota composition in 6 month-old offspring**

The RDA analysis showed no significant effects of maternal ATB treatment and offspring late diet on offspring colonic microbiota composition (data not shown).

**Colonic crypt architecture**

Colonic crypt architecture of pigs in the LT trial was not influenced by treatments, except for a trend (P = 0.067) for a small numerical difference (4%) between treatments (ATB > CTL) in crypt width (S4 Table).

**Colonic permeability and electrophysiology (Ussing chambers)**

Colonic permeability to FD4 in basal condition displayed a highly significant interaction between maternal ATB treatment and offspring late diet (P < 0.01) (Table 2). In CTL offspring, FD4 permeability was lower when fed the HF compared to the LF diet (P < 0.05) while this difference was not significant in ATB offspring. Under oxidative condition, colonic permeability to FD4 tended (P = 0.10) to be higher in ATB offspring compared to CTL. In this case, the treatment by diet interaction was not significant.

**Table 2:** Mucosal-to-serosal fluxes of FD4 and HRP for basal and monochloramine-stimulated colonic mucosa of pigs born to control or antibiotic-treated sows and fed a low (LF) or a high (HF) fat diet between 140 and 169 days of age (LSmeans and SEM, n = 8–10 per treatment).

*Sow’s treatment*	**Control**		**Antibiotics**			**Statistics (P =)** ^**1**^		
*Offspring’s diet*	**LF**	**HF**	**LF**	**HF**	**SEM**	**treat**.	**diet**	treat.*diet
**FD4**								
FD4, basal (ng/cm²/h)	1310^a^	726^b^	851^b^	1057^a^ ^b^	142	0.65	0.33	0.009
FD4, + monochloramine (% basal)	92	137	139	148	17	0.10	0.15	0.26
**HRP**								
HRP, basal (ng/cm²/h)	158	117	187	169	47	0.37	0.48	0.80
HRP, + monochloramine (% basal)	261	140	116	105	41	0.047	0.20	0.18

^1^Treat.: Treatment of sows pre- and post-partum (control *versus* antibiotic); diet (low *versus* high fat diet); treat.*diet: treatment by diet interaction.

^a, b^ LS means with different superscript letters in a row differ (P<0.05).

Colonic permeability to HRP in basal condition was not influenced by maternal treatment or offspring diet. However, under oxidative condition, permeability to HRP was lower in ATB offspring than in CTL (P = 0.047) (Table 2). The treatment by diet interaction was never significant.

Offspring colonic electrophysiology was not influenced by maternal ATB treatment or offspring late diet, except for glucose-induced change in Isc in oxidative condition that tended (P = 0.087) to be higher in offspring born to ATB mothers than in CTL (S5 Table). Diet effects and interactions were not significant.

**Colonic heat shock proteins**

Protein concentrations of HSP27 and HSP70 (relative to β-actin) in colonic tissues did not display any significant effects of maternal ATB treatment or offspring diet, or significant treatment by diet interactions (S1 Fig.). Therefore, protein concentrations of HSF1 in colonic tissues were not determined in the LT experiment.

**AP activity concentration in colonic tissues and digesta contents**

AP activity concentrations in colonic tissues were not influenced by maternal ATB treatment or offspring late diet, and the treatment by diet interaction was not significant (Table 3). By contrast, maternal treatment was (P = 0.037) (or tended to be, P = 0.097, depending on the mode of expression of data) significant for colonic digesta AP concentration, and the treatment by diet interaction was significant (P < 0.05 and P < 0.01, for data expressed per weight of fresh digesta and g of soluble protein, respectively) (Table 3). Colonic digesta AP was 80% higher (P < 0.05) in ATB offspring consuming the HF diet, compared to the other groups.

**Table 3:** Alkaline phosphatase (AP) activity concentrations in colonic tissue and digesta contents of pigs born to control or antibiotic-treated sows and fed a low (LF) or a high (HF) fat diet between 140 and 169 days of age (LSmeans and SEM, n = 10 per treatment).

*Sow’s treatment*	**Control**		**Antibiotics**			**Statistics (P =)** ^**1**^		
*Offspring’s diet*	**LF**	**HF**	**LF**	**HF**	**SEM**	**treat**.	**diet**	treat.*diet
**Colonic tissue**								
μg AP/ g tissue	4.73	4.47	5.34	4.48	0.78	0.71	0.47	0.70
μg AP/ g protein	117	106	131	121	17	0.43	0.52	0.97
**Colonic digesta**								
μg AP/ g fresh digesta	17^b^	14^b^	18^b^	30^a^	3	0.037	0.37	0.042
μg AP/ g protein	1.06^a^ ^b^	0.73^b^	0.85^b^	1.55^a^	0.17	0.097	0.27	0.005

^1^Treat.: Treatment of sows pre- and post-partum (control *versus* antibiotic); diet (low *versus* high fat diet); treat.*diet: treatment by diet interaction.

^a, b^ LS means with different superscript letters in a row differ (P<0.05).

**Concentrations of TLR2- and TLR4- stimulants in feed and rectal contents**

**Fig 3:**
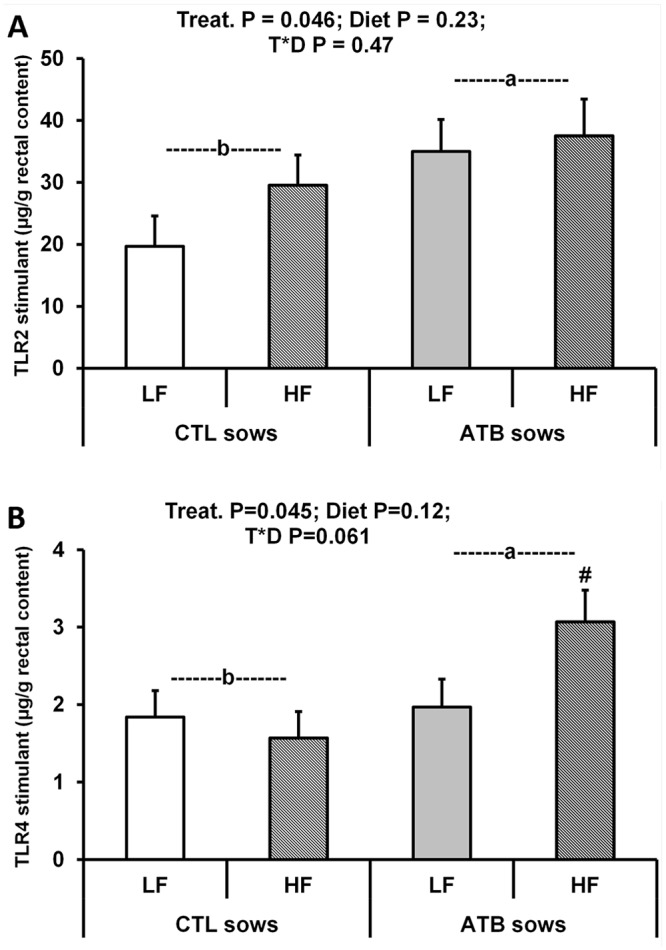
Rectal TLR-stimulant concentrations in fresh rectal contents of offspring born to control or antibiotic-treated sows and fed a LF or a HF diet between 140 and 169 days of age (LSmeans and SEM, n = 10 per treatment and diet). **A.** TLR2-stimulant concentration was significantly higher (P < 0.05) in fresh rectal contents of offspring born to ATB sows compared to controls when fed the LF diet. **B.** Rectal TLR4-stimulant concentration was significantly higher (P < 0.05) in fresh rectal contents of offspring born to ATB sows and fed the LF diet, compared to the other groups (treatment by diet interaction, P = 0.061; LSmean with # tended to be different from the others, P < 0.10).

The concentrations of TLR-stimulants amounted to 1772 and 1322 μg/g for TLR2-stimulants, and 924 and 1275 μg/g for TLR4-stimulants, in LF and HF feed, respectively. The concentrations of TLR2- and TLR4- stimulants in fresh rectal contents were higher in offspring born to ATB-treated sows than in CTL offspring (P < 0.05) (Fig. 3A and 3B). The diet effect and the treatment by diet interaction were not significant for TLR2-stimulants, but the interaction tended towards significance for TLR4-stimulants (P = 0.061). Rectal concentration of TLR4-stimulants tended to be higher in offspring born to ATB-treated sows and fed the HF diet than in the other groups (Fig. 3B, P < 0.10). Expressing TLR-stimulant concentrations on a dry matter basis confirmed the results obtained with fresh rectal contents since dry matter content was not influenced by the tested factors (S6 Table).

## Discussion

We report important data on short-term and long-term effects of early changes in microbial colonization on colonic physiology in a swine model of maternal antibiotic treatment around parturition. We show that this treatment increased inducible HSP proteins consistently in colonic tissue, and increased transiently HRP permeability under oxidative stress in the ST trial in offspring born to ATB-treated mothers. The long term effects on the colon were characterized by lower colonic HRP permeability under oxidative stress and higher concentrations of AP activity and TLR-stimulants in colonic and rectal contents, respectively. Importantly, late HF diet also disclosed interactions with early treatment on a number of colonic parameters (permeability, AP activity concentration, rectal TLR-stimulants). Conversely, many variables (e.g HSPs in the long-term study) were not influenced by early mother treatment (or interaction), illustrating the selectivity of early programming of colonic functions.

### The pig model: effects of maternal ATB treatment on offspring colonic microbiota

Changes in colonic microbiota composition appeared limited in this study, as already mentioned for ileal microbiota, and may suggest that our model of maternal antibiotic induction of disturbances in offspring gut colonization is rather mild and physiological [20]. This is in sharp contrast with the dramatic effects observed on the gut microbiota when amoxicillin was directly administered orally or intramuscularly to rodent or pig offspring [19,31,32]. Nevertheless, some changes in the relative contribution of various bacterial genera and species were recorded here, and one cannot exclude the possibility that microbiota metabolic activities were also modified. Unfortunately, we did not look to that aspect in the present work. The consequences of these bacterial changes on colonic physiology are difficult to interpret in a simple way as they occurred simultaneously. But it is noteworthy mentioning that some species (e.g. *Lactobacillus plantarum*) or mixtures (e.g. VSL#3 mixture, containing, among others: *L*. *plantarum*, *L*. *acidophilus*, *L*. *delbrueckii*) of some of these bacteria can modulate gut epithelial physiology as shown with rodent cell cultures [13,33,34]. Other bacteria may have opposite effects. The underlying mechanisms of action are sometimes unknown, but they may involve various bacterial factors (e.g. bacterial components, secreted molecules or fermentation products) and signaling pathways [33].

Although one cannot exclude the possibility that a small fraction of amoxicillin given orally to sows would actually be transferred to their offspring through colostrum and milk, and act directly on offspring microbiota, this might be very limited for the following reasons. First, the present data indicate a very limited impact of maternal amoxicillin treatment on offspring colonic microbiota composition. Second, pharmacokinetic studies carried out in growing pigs (25 kg BW) with an oral bolus of amoxicillin (20 mg/kg BW) revealed low bioavailability (22.8%) and very short plasma elimination half-life (0.73 h) [35]. Third, we previously showed that single intramuscular injection of amoxicillin (15 mg/kg BW) to one day-old piglets profoundly disturbed their colonic microbiota ecosystem as investigated 38 days later (final BW: 11 kg) [31]. Therefore, for all these reasons, we can reasonably suggest that the direct action of maternally provided amoxicillin on offspring microbiota may be very limited in the present study.

### Short-term influence of maternal ATB administration on offspring colonic physiology

**Inducible HSPs**

The major finding of the present work is that early changes in offspring gut bacterial colonization, as induced by maternal ATB administration peripartum led to a twofold increase in both HSP27 and HSP70 levels in offspring colonic tissue consistently between 14 and 42 days of age. This may mirror subtle changes in microbiota composition or activity as luminal bacteria (e.g. *E*. *coli*, *Bacillus fragilis*) [10], bacterial components (e.g. LPS, flagellin) [11,13], and fermentation products (e.g. propionate and butyrate) [9] have been reported to induce protective HSPs specifically in colonic epithelial cells. This induction involves MyD88 and TLR-dependent signaling pathways [11,14]. We previously reported in the present model that maternal ATB treatment actually decreased offspring intestinal HSP70 with no effect on HSP27 in the small intestine [20]. Collectively, these data suggest a higher bacterial stimulation in the colon, and a lower one in the small intestine of pigs born to antibiotic-treated sows. They also suggest site-dependent differential responses between HSP27 and HSP70. Importantly, a direct link was recently revealed between colonic HSP levels and richness and diversity of the microbiota in close contact with the colonic mucosa in mice [36]. There was no significant correlation between microbiota diversity composition and colonic HSP levels in the present study, possibly because it was the luminal microbiota that we analyzed. Indeed, a recent study in pigs disclosed more frequent correlations between (intestinal or colonic) inducible HSPs and mucosal than with luminal microbiota [37]. Beside, inducible HSPs are major protective factors for the gut epithelium submitted to various stressors, including oxidation and inflammation. The fact that we did not evidence differences between treatment groups in colonic responsiveness to oxidative stress in Ussing chambers may indicate that higher HSP levels in ATB offspring did not apparently confer more protection than that observed in CTL offspring. HSF1 is a key transcriptional regulator of HSP gene expression [38]. The level of HSF1 protein expression did not vary significantly in colonic tissues, as reported for ileal tissues [20]. Our data thus suggest a post-translational regulation of intracellular HSP concentration that may reflect for example differences in HSP degradation pathways (e.g. ubiquitination) [39,40]. These mechanisms could have been downregulated in offspring born to ATB-treated mothers.

**Colonic permeability and electrophysiology**

Our study with the oxidant monochloramine revealed higher transcellular permeability in ATB offspring transiently at 14 days of age. This may have impacted the education of the underlying immune system in developing offspring [41], but we did not investigate this question.

### Long-term influence of maternal ATB treatment on offspring colonic physiology

**Colonic permeability**

Colonic permeability under oxidative condition was influenced by maternal ATB treatment. HRP permeability was lower in ATB offspring. Molecular mechanisms of gut permeability are quite complex but increased permeability is often associated with mucosal inflammation and chronic diseases [41]. In the present swine model, we noted a higher plasma concentration of α-acid glycoprotein in adult ATB offspring [20]. This finding may suggest less systemic inflammation in these ATB offspring as plasma α-acid glycoprotein decreases in many models of aseptic inflammation or following infections [42]. Increased paracellular permeability has been ascribed to reduced levels of epithelial HSPs in some studies [26,43], but we did not see any treatment effect on inducible HSPs in the present LT trial.

As HRP crossing the epithelium is essentially trans-cellular under non-stimulated conditions [28], the decreased HRP permeability observed here suggests partial reduction in colonic cell protein endocytosis [41]. Inhibition of transcellular transport has been shown to involve various functional proteins (e.g. caveolin-1, dynamin and intersectins) and, more importantly, to increase paracellular permeability in lung epithelium [44]. This inverse relationship between trans- and para-cellular permeabilities may account for our observations. Another possible mechanism may be lower colonic stimulation by corticotropin-releasing factor (CRF) because this neuro-immune mediator is known to specifically stimulate trans-cellular uptake of macromolecules in human colon [45].

Colonic tissue and digesta AP activities

Colonic tissue AP activity is a marker of inflammation [46]. Oxidative stress up-regulates colonic tissue non-specific AP (TNAP) and neutrophils infiltrating colonic tissues also express the TNAP isoform [46,47]. Thus, colonic tissue inflammation was equally low on both treatments groups in the present study. By contrast, we found colonic digesta AP activity concentration to be much higher in adult ATB offspring compared to controls. Colonic AP activity may reasonably come from undigested (but still active) AP enzyme from the small intestine as it is an enterocyte-secreted enzyme protein resisting partially intestinal digestion and colonic fermentation [48,49]. In the small intestine, we observed treatment differences in jejunal (but not ileal) tissue AP activity concentrations [20], but this was surprisingly not reflected in ileal digesta AP activity concentrations (J.P. Lallès, unpublished data). As quantitative digesta flows were not measured here, we can only suggest that colonic microbiota proteolytic activity towards AP enzyme may have been probably higher in adult ATB offspring than in controls. The actual reasons for this are presently unknown.

**Bioactive TLR-stimulants in rectal contents**

An original aspect of the present works is that we show early maternal ATB administration to impact on rectal concentrations of bioactive MAMPs in ATB offspring in adulthood. This is important because MAMPs such as LPS, once having entered the body at the gut level (e.g. after chronic consumption of a HF diet), are strongly suspected to induce low-grade, metabolic inflammation and be causally responsible for the development of metabolic diseases and obesity [5]. Indeed, particular TLRs such as TLR2 and TLR4 link inflammation to metabolism and obesity [6,7].

Chemical-induced gut inflammation dramatically increased colonic concentrations of lipopeptide (ligand of TLR2; fourfold) and LPS in mice (ligand for TLR4; 550-fold) [15]. This reflected increased colonic concentrations of enterobacterial species rather than Bacteroides or Gram-positive Firmicutes [15]. Here, we observed variations in TLR-stimulant concentrations that were more reasonable (1.5 to twofold). Of note, we were able to discriminate between early treatments: ATB offspring displayed higher rectal concentrations of TLR2- and TLR4- stimulants than CTL offspring. Although the physiological meaning of our observations is not known yet, it may reflect differences in bacterial production and/or degradation of TLR-stimulants in the large intestine. Indeed, concentrations of TLR2- and TLR4- stimulants in the ileum did not differ between CTL and ATB treatments or between diets (data not shown). Finally, these differences in rectal bioactive MAMP concentrations did not seem to reflect differences in digesta AP activities (and MAMP-detoxifying capacity) in the proximal colon.

### Dietary modulation of functional responses of the colon in adult offspring

Our data show that some colonic functions programmed early in life may be revealed only under challenge with an unbalanced (HF) diet consumed later in life. These include colonic basal HRP permeability, AP activity concentration in colonic digesta, and rectal concentrations of TLR4-stimulants to some extent.

Surprisingly, colonic basal paracellular permeability was found to be lower in CTL offspring fed the LF compared to the HF diet. HF diet increased colonic paracellular permeability in rats, an effect mediated through bile acids [50]. Unfortunately, we did not find published data on the effects of palm oil on colonic barrier function. A study in pigs suggested that acute consumption of saturated (but medium chain, coconut oil) fat increased plasma LPS concentrations compared to a poly-unsaturated fatty acid-containing test diet [51]. Plasma LPS mirrors gut permeability [5]. Here, plasma levels of haptoglobin, a marker of inflammation, tended to be higher with the HF diet, but with no differences between CTL and ATB offspring [20]. Nevertheless, our data show that this difference between LF and HF diets in CTL offspring completely vanished in offspring born to ATB-treated sows, suggesting an influence of maternal ATB treatment peripartum on offspring colonic paracellular permeability later in life. This clearly needs more specific investigations.

AP activity concentration in colonic digesta was twofold higher in ATB offspring fed the HF diet compared to the other groups. This might suggest that HF intake may have stimulated AP protein fermentation in the colon of ATB offspring. In a recent study in pigs, an HF diet (17% fat provided by lard) was reported to decrease cecal fermentation, with no effects on microbiota diversity composition [52]. Reasons for these apparent discrepancies and underlying mechanisms are presently unknown. Rectal concentration of TLR4-stimulants also tended to be higher in ATB offspring fed the HF diet compared to the other groups, suggesting the possibility of a higher bacterial production and/or a lower detoxification of these TLR-stimulants in this ATB-HF group. This would be consistent with previous observations on digesta TLR-stimulant concentrations in mice fed a very high fat diet [16].

## Conclusions and Perspectives

We developed a swine model of mild neonatal changes in microbial colonization induced by antibiotic treatment of dams around parturition and we showed that this treatment influenced selectively some aspects of colonic physiology in the short-term, including a large increase in tissue levels of inducible HSPs. More importantly, our data support the notion that these early changes in gut microbial colonization might have long-term influences on various facets of colonic physiology especially under stressful condition, although these changes did not seem to be directly related to late colonic microbiota composition. This area warrants further investigation on the underlying mechanisms of early colonic programming.

## Supporting Information

S1 Fig Protein expression of heat shock proteins in colonic tissues of pigs born to control or antibiotic-treated sows and fed a low (LF) or a high (HF) fat diet between 140 and 169 days of age (LSmeans and SEM, n = 8–10 per treatment and diet). **A.** HSP27. **B.** HSP70.
(TIF)

S1 Table Crypt architecture of colonic mucosa in pigs born to control or antibiotic-treated sows and slaughtered at different ages (LSmeans and SEM, n = 11–12 per treatment and age). (DOCX)

S2 Table Electrophysiological characteristics of basal and monochloramine-stimulated colonic mucosa of pigs born to control or antibiotic-treated sows and slaughtered at different ages (LSmeans and SEM, n = 5–6 per treatment and age). (DOCX)

S3 Table Alkaline phosphatase (AP) activity concentrations in colonic mucosa and in cecal and rectal digesta contents of pigs born to control or antibiotic-treated sows and slaughtered at different ages (LSmeans and SEM, n = 10–12 per treatment and age). (DOCX)

S4 Table Crypt architecture of colonic mucosa in pigs born to control or antibiotic-treated sows and fed a low (LF) or a high (HF) fat diet between 140 and 169 days of age (LSmeans and SEM, n = 10 per treatment and diet). (DOCX)

S5 Table Electrophysiological characteristics of basal and monochloramine-stimulated colonic mucosa of pigs born to control or antibiotic-treated sows and fed a low (LF) or a high (HF) fat diet between 140 and 169 days of age (LSmeans and SEM, n = 8–10 per treatment and diet). (DOCX)

S6 Table Concentrations of TLR-stimulants in rectal contents of pigs born to control or antibiotic-treated sows and fed a low (LF) or a high (HF) fat diet between 140 and 169 days of age (LSmeans and SEM, n = 8–10 per treatment and diet). (DOCX)

## References

[1] Ruel J, Ruane D, Mehandru S, Gower-Rousseau C, Colombel JF (2014) IBD across the age spectrum-is it the same disease? Nat Rev Gastroenterol Hepatol 11: 88–98. 10.1038/nrgastro.2013.240 24345891

[2] Farkas K, Yeruva S, Rakonczay Z Jr, Ludolph L, Molnar T, et al (2011) New therapeutic targets in ulcerative colitis: the importance of ion transporters in the human colon. Inflamm Bowel Dis 17: 884–898. 10.1002/ibd.21432 20722063

[3] Pastorelli L, De Salvo C, Mercado JR, Vecchi M, Pizarro TT (2013) Central role of the gut epithelial barrier in the pathogenesis of chronic intestinal inflammation: lessons learned from animal models and human genetics. Front Immunol 4: 280 10.3389/fimmu.2013.00280 24062746 PMC3775315

[4] Keely S, Kelly CJ, Weissmueller T, Burgess A, Wagner BD, et al (2012) Activated fluid transport regulates bacterial-epithelial interactions and significantly shifts the murine colonic microbiome. Gut Microbes 3: 250–260. 10.4161/gmic.20529 22614705 PMC3427217

[5] Cani PD, Delzenne NM (2009) The role of the gut microbiota in energy metabolism and metabolic disease. Curr Pharm Des 15: 1546–1558. 19442172 10.2174/138161209788168164

[6] Konner AC, Bruning JC (2011) Toll-like receptors: linking inflammation to metabolism. Trends Endocrinol Metab 22: 16–23. 10.1016/j.tem.2010.08.007 20888253

[7] Jin C, Flavell RA (2013) Innate sensors of pathogen and stress: linking inflammation to obesity. J Allergy Clin Immunol 132: 287–294. 10.1016/j.jaci.2013.06.022 23905917

[8] Sommer F, Backhed F (2013) The gut microbiota—masters of host development and physiology. Nat Rev Microbiol 11: 227–238. 10.1038/nrmicro2974 23435359

[9] Ren H, Musch MW, Kojima K, Boone D, Ma A, et al (2001) Short-chain fatty acids induce intestinal epithelial heat shock protein 25 expression in rats and IEC 18 cells. Gastroenterology 121: 631–639. 11522747 10.1053/gast.2001.27028

[10] Kojima K, Musch MW, Ren H, Boone DL, Hendrickson BA, et al (2003) Enteric flora and lymphocyte-derived cytokines determine expression of heat shock proteins in mouse colonic epithelial cells. Gastroenterology 124: 1395–1407. 12730879 10.1016/s0016-5085(03)00215-4

[11] Kojima K, Musch MW, Ropeleski MJ, Boone DL, Ma A, et al (2004) Escherichia coli LPS induces heat shock protein 25 in intestinal epithelial cells through MAP kinase activation. Am J Physiol Gastrointest Liver Physiol 286: G645–652. 14630641 10.1152/ajpgi.00080.2003

[12] Petrof EO, Ciancio MJ, Chang EB (2004) Role and regulation of intestinal epithelial heat shock proteins in health and disease. Chin J Dig Dis 5: 45–50. 15612656 10.1111/j.1443-9573.2004.00154.x

[13] Petrof EO, Musch MW, Ciancio M, Sun J, Hobert ME, et al (2008) Flagellin is required for salmonella-induced expression of heat shock protein Hsp25 in intestinal epithelium. Am J Physiol Gastrointest Liver Physiol 294: G808–818. 10.1152/ajpgi.00362.2007 18202113

[14] Rakoff-Nahoum S, Paglino J, Eslami-Varzaneh F, Edberg S, Medzhitov R (2004) Recognition of commensal microflora by toll-like receptors is required for intestinal homeostasis. Cell 118: 229–241. 15260992 10.1016/j.cell.2004.07.002

[15] Erridge C, Duncan SH, Bereswill S, Heimesaat MM (2010) The induction of colitis and ileitis in mice is associated with marked increases in intestinal concentrations of stimulants of TLRs 2, 4, and 5. PLoS ONE 5: e9125 10.1371/journal.pone.0009125 20161736 PMC2817728

[16] Kim KA, Gu W, Lee IA, Joh EH, Kim DH (2012) High fat diet-induced gut microbiota exacerbates inflammation and obesity in mice via the TLR4 signaling pathway. PLoS One: 7: e47713 10.1371/journal.pone.0047713 23091640 PMC3473013

[17] Lalles JP (2012) Long term effects of pre- and early postnatal nutrition and environment on the gut. J Anim Sci 90 Suppl 4: 421–429. 23365399 10.2527/jas.53904

[18] Fanca-Berthon P, Hoebler C, Mouzet E, David A, Michel C (2010) Intrauterine growth restriction not only modifies the cecocolonic microbiota in neonatal rats but also affects its activity in young adult rats. J Pediatr Gastroenterol Nutr 51: 402–413. 10.1097/MPG.0b013e3181d75d52 20601908

[19] Schumann A, Nutten S, Donnicola D, Comelli EM, Mansourian R, et al (2005) Neonatal antibiotic treatment alters gastrointestinal tract developmental gene expression and intestinal barrier transcriptome. Physiol Genomics 23: 235–245. 16131529 10.1152/physiolgenomics.00057.2005

[20] Arnal ME, Zhang J, Messori S, Bosi P, Smidt H, et al (2014) Early changes in microbial colonization selectively modulate intestinal enzymes, but not inducible heat shock proteins in young adult swine. PLoS ONE: 9(2):e87967 10.1371/journal.pone.0087967 24505340 PMC3913709

[21] Lalles JP, Orozco-Solis R, Bolanos-Jimenez F, de Coppet P, Le Drean G, et al (2012) Perinatal undernutrition alters intestinal alkaline phosphatase and its main transcription factors KLF4 and Cdx1 in adult offspring fed a high-fat diet. J Nutr Biochem 23: 1490–1497. 10.1016/j.jnutbio.2011.10.001 22405696

[22] Perez Gutierrez O (2010) Unraveling piglet gut microbiota dynamics in response to feed additives [PhD thesis]. Wageningen: Wageningen University, The Netherlands 198 p.

[23] Haenen D, Zhang J, Souza da Silva C, Bosch G, van der Meer IM, et al (2013) A diet high in resistant starch modulates microbiota composition, SCFA concentrations, and gene expression in pig intestine. J Nutr 143: 274–283. 10.3945/jn.112.169672 23325922

[24] Le Gall M, Gallois M, Seve B, Louveau I, Holst JJ, et al (2009) Comparative effect of orally administered sodium butyrate before or after weaning on growth and several indices of gastrointestinal biology of piglets. Br J Nutr 102: 1285–1296. 10.1017/S0007114509990213 19480733

[25] Lalles JP, Boudry G, Favier C, Seve B (2006) High-viscosity carboxymethylcellulose reduces carbachol-stimulated intestinal chloride secretion in weaned piglets fed a diet based on skimmed milk powder and maltodextrin. Br J Nutr 95: 488–495. 16512934 10.1079/bjn20051673

[26] Arvans DL, Vavricka SR, Ren H, Musch MW, Kang L, et al (2005) Luminal bacterial flora determines physiological expression of intestinal epithelial cytoprotective heat shock proteins 25 and 72. Am J Physiol Gastrointest Liver Physiol 288: G696–704. 15528251 10.1152/ajpgi.00206.2004

[27] Chatelais L, Jamin A, Gras-Le Guen C, Lalles JP, Le Huerou-Luron I, et al (2011) The level of protein in milk formula modifies ileal sensitivity to LPS later in life in a piglet model. PLoS ONE 6: e19594 10.1371/journal.pone.0019594 21573022 PMC3090415

[28] Wijtten PJ, van der Meulen J, Verstegen MW (2011) Intestinal barrier function and absorption in pigs after weaning: a review. Br J Nutr 105:967–981. 10.1017/S0007114510005660 21303573

[29] David JC, Grongnet JF, Lalles JP (2002) Weaning affects the expression of heat shock proteins in different regions of the gastrointestinal tract of piglets. J Nutr 132: 2551–2561. 12221208 10.1093/jn/132.9.2551

[30] Van den Brink PJ, Cajo JF, Braak T (1999). Principal response curves: Analysis of time-dependent multivariate responses of biological community to stress. Env Tox Chem 18:138–148.

[31] Janczyk P, Pieper R, Souffrant WB, Bimczok D, Rothkotter HJ, et al (2007) Parenteral long-acting amoxicillin reduces intestinal bacterial community diversity in piglets even 5 weeks after the administration. ISME J 1: 180–183. 18043627 10.1038/ismej.2007.29

[32] Fak F, Ahrne S, Molin G, Jeppsson B, Westrom B (2008) Microbial manipulation of the rat dam changes bacterial colonization and alters properties of the gut in her offspring. Am J Physiol Gastrointest Liver Physiol 294: G148–154. 17962363 10.1152/ajpgi.00023.2007

[33] Lebeer S, Vanderleyden J, De Keersmaecker SC (2008) Genes and molecules of lactobacilli supporting probiotic action. Microbiol Mol Biol Rev 72: 728–764. 10.1128/MMBR.00017-08 19052326 PMC2593565

[34] Petrof EO, Claud EC, Sun J, Abramova T, Guo Y, et al (2009) Bacteria-free solution derived from Lactobacillus plantarum inhibits multiple NF-kappaB pathways and inhibits proteasome function. Inflamm Bowel Dis 15: 1537–1547. 10.1002/ibd.20930 19373789 PMC2748164

[35] Reyns T, De Boever S, Baert K, Croubels S, Schauvliege S, et al (2007) Disposition and oral bioavailability of amoxicillin and clavulanic acid in pigs. J Vet Pharmacol Ther 30: 550–555. 17991223 10.1111/j.1365-2885.2007.00910.x

[36] Hu S, Wang Y, Lichtenstein L, Tao Y, Musch MW, et al (2010) Regional differences in colonic mucosa-associated microbiota determine the physiological expression of host heat shock proteins. Am J Physiol Gastrointest Liver Physiol 299: G1266–1275. 10.1152/ajpgi.00357.2010 20864653 PMC3006241

[37] Liu HY, Dicksved J, Lundh T, Lindberg JE (2014) Expression of heat shock proteins 27 and 72 correlates with specific commensal microbes in different regions of porcine gastrointestinal tract. Am J Physiol Gastrointest Liver Physiol 306: G1033–1041. 10.1152/ajpgi.00299.2013 24763551

[38] Westerheide SD, Raynes R, Powell C, Xue B, Uversky VN (2012) HSF transcription factor family, heat shock response, and protein intrinsic disorder. Curr Protein Pept Sci 13: 86–103. 22044151 10.2174/138920312799277956

[39] Qian SB, McDonough H, Boellmann F, Cyr DM, Patterson C (2006) CHIP-mediated stress recovery by sequential ubiquitination of substrates and Hsp70. Nature 440: 551–555. 16554822 10.1038/nature04600 PMC4112096

[40] Voellmy R, Boellmann F (2007) Chaperone regulation of the heat shock protein response. Adv Exp Med Biol 594: 89–99. 17205678 10.1007/978-0-387-39975-1_9

[41] Menard S, Cerf-Bensussan N, Heyman M (2010) Multiple facets of intestinal permeability and epithelial handling of dietary antigens. Mucosal Immunol 3: 247–259. 10.1038/mi.2010.5 20404811

[42] Heegaard PM, Miller I, Sorensen NS, Soerensen KE, Skovgaard K (2013) Pig alpha1-acid glycoprotein: characterization and first description in any species as a negative acute phase protein. PLoS ONE 8: e68110 10.1371/journal.pone.0068110 23844161 PMC3699587

[43] Musch MW, Sugi K, Straus D, Chang EB (1999) Heat-shock protein 72 protects against oxidant-induced injury of barrier function of human colonic epithelial Caco2/bbe cells. Gastroenterology 117: 115–122. 10381917 10.1016/s0016-5085(99)70557-3

[44] Van Driessche W, Kreindler JL, Malik AB, Margulies S, Lewis SA, et al (2007) Interrelations/cross talk between transcellular transport function and paracellular tight junctional properties in lung epithelial and endothelial barriers. Am J Physiol Lung Cell Mol Physiol 293: L520–524. 17601795 10.1152/ajplung.00218.2007

[45] Wallon C, Soderholm JD (2009) Corticotropin-releasing hormone and mast cells in the regulation of mucosal barrier function in the human colon. Ann N Y Acad Sci 1165: 206–210. 10.1111/j.1749-6632.2009.04030.x 19538308

[46] Lalles JP (2014) Intestinal alkaline phosphatase: novel functions and protective effects. Nutr Rev 72: 82–94. 10.1111/nure.12082 24506153

[47] Lopez-Posadas R, Gonzalez R, Ballester I, Martinez-Moya P, Romero-Calvo I, et al (2011) Tissue-nonspecific alkaline phosphatase is activated in enterocytes by oxidative stress via changes in glycosylation. Inflamm Bowel Dis 17: 543–556. 10.1002/ibd.21381 20645320

[48] Goldberg RF, Austen WG, Zhang XB, Munene G, Mostafa G, et al (2008) Intestinal alkaline phosphatase is a gut mucosal defense factor maintained by enteral nutrition. Proc Natl Acad Sci U S A 105: 3551–3556. 10.1073/pnas.0712140105 18292227 PMC2265168

[49] Lalles JP (2010) Intestinal alkaline phosphatase: multiple biological roles in maintenance of intestinal homeostasis and modulation by diet. Nutr Rev 68: 323–332. 10.1111/j.1753-4887.2010.00292.x 20536777

[50] Stenman LK, Holma R, Korpela R (2012) High-fat-induced intestinal permeability dysfunction associated with altered fecal bile acids. World J Gastroenterol 18: 923–929. 10.3748/wjg.v18.i9.923 22408351 PMC3297051

[51] Mani V, Hollis JH, Gabler NK (2013) Dietary oil composition differentially modulates intestinal endotoxin transport and postprandial endotoxemia. Nutr Metab 10: 6 10.1186/1743-7075-10-6 23305038 PMC3577458

[52] Yan H, Potu R, Lu H, Vezzoni de Almeida V, Stewart T, et al (2013) Dietary fat content and fiber type modulate hind gut microbial community and metabolic markers in the pig. PLoS ONE 8: e59581 10.1371/journal.pone.0059581 23573202 PMC3616062

