# Supplementary figures and images for: Maternal Antibiotic-Induced Early Changes in Microbial Colonization Selectively Modulate Colonic Permeability and Inducible Heat Shock Proteins, and Digesta Concentrations of Alkaline Phosphatase and TLR-Stimulants in Swine Offspring
Source: PLoS One. 2015 Feb 17;10(2):e0118092. doi: 10.1371/journal.pone.0118092 (PMC4331088; doi:10.1371/journal.pone.0118092)

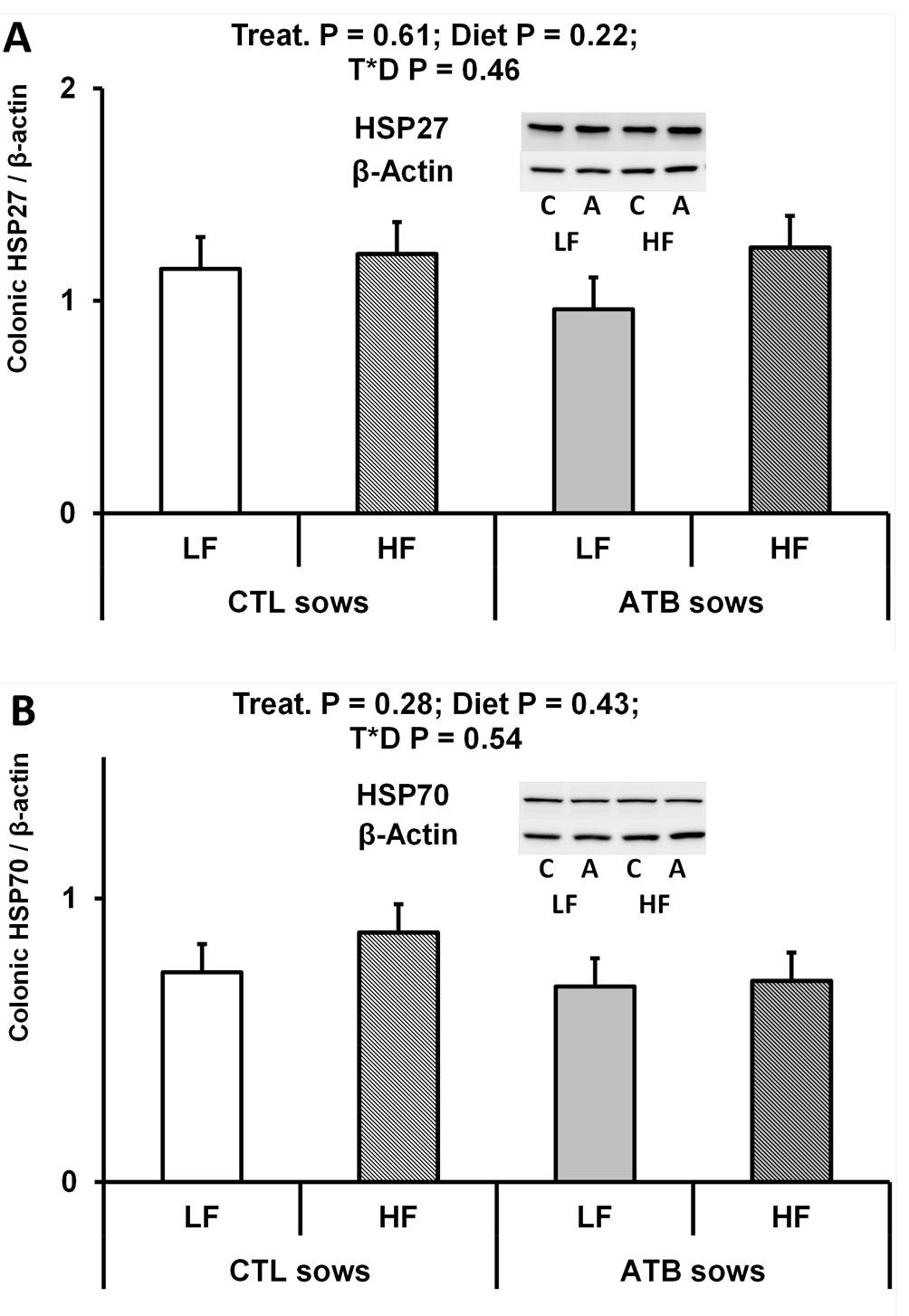

Supplement: S1 Fig — A. HSP27. B. HSP70. (TIF) [file pone.0118092.s001.tif]
