# Supplementary material for: Maternal Antibiotic-Induced Early Changes in Microbial Colonization Selectively Modulate Colonic Permeability and Inducible Heat Shock Proteins, and Digesta Concentrations of Alkaline Phosphatase and TLR-Stimulants in Swine Offspring
Source: PLoS One. 2015 Feb 17;10(2):e0118092. doi: 10.1371/journal.pone.0118092 (PMC4331088; doi:10.1371/journal.pone.0118092)
Supplement: S1 Table — (DOCX) [file pone.0118092.s002.docx]

**Table S1. Crypt architecture of colonic mucosa in pigs born to control or antibiotic-treated sows and slaughtered at different ages (LSmeans and SEM, n = 11-12 per treatment and age).**

| *Sow’s treatment* | **Control** | | |  | **Antibiotic** | | |  |  |  | **Statistics (P =)^1^** | |  |
| --- | --- | --- | --- | --- | --- | --- | --- | --- | --- | --- | --- | --- | --- |
| *Offspring’s age* | **d14** | **d28** | **d42** |  | **d14** | **d28** | **d42** |  | **SEM** |  | **treat.** | **age** | **treat.*age** |
| Depth (µm) | 272 | 306 | 293 |  | 269 | 327 | 257 |  | 18 |  | 0.72 | 0.031 | 0.31 |
| Width (µm) | 65.1 | 63.4 | 73.7 |  | 65.6 | 63.1 | 72.5 |  | 2.3 |  | 0.85 | 0.0004 | 0.94 |
| Perimeter (µm) | 643 | 713 | 695 |  | 638 | 752 | 624 |  | 38 |  | 0.71 | 0.049 | 0.45 |
| Surface area (µm² x 10^3^) | 16.0 | 17.8 | 18.9 |  | 16.0 | 18.6 | 16.4 |  | 1.4 |  | 0.63 | 0.26 | 0.45 |

^1^Treat.: Treatment of sows pre- and post-partum (control *versus* antibiotic); age (d14 and d28, unweaned; d42 weaned from d28); treat.*age: treatment by age interaction.
