# Supplementary material for: Maternal Antibiotic-Induced Early Changes in Microbial Colonization Selectively Modulate Colonic Permeability and Inducible Heat Shock Proteins, and Digesta Concentrations of Alkaline Phosphatase and TLR-Stimulants in Swine Offspring
Source: PLoS One. 2015 Feb 17;10(2):e0118092. doi: 10.1371/journal.pone.0118092 (PMC4331088; doi:10.1371/journal.pone.0118092)
Supplement: S2 Table — (DOCX) [file pone.0118092.s003.docx]

**Table S2. Electrophysiological characteristics of basal and monochloramine-stimulated colonic mucosa of pigs born to control or antibiotic-treated sows and slaughtered at different ages (LSmeans and SEM, n = 5-6 per treatment^1^).**

| *Sow’s treatment* | **Control** | | |  | **Antibiotic** | | |  |  |  | **Statistics (P =)^1^** | |  |
| --- | --- | --- | --- | --- | --- | --- | --- | --- | --- | --- | --- | --- | --- |
| *Offspring’s age* | **d14** | **d28** | **d42** |  | **d14** | **d28** | **d42** |  | **SEM** |  | **treat.** | **age** | **treat.*age** |
| **Basal condition** |  |  |  |  |  |  |  |  |  |  |  |  |  |
| Isc (µA/cm²)^3^ | 79 | 14 | 21 |  | 75 | 53 | 42 |  | 26 |  | 0.41 | 0.17 | 0.71 |
| TEER (Ω x cm²)^4^ | 45 | 66 | 49 |  | 46 | 43 | 42 |  | 7 |  | 0.16 | 0.43 | 0.30 |
| ΔIsc, glucose (µA/cm²)^5^ | 3.2 | 1.6 | 0.4 |  | 10.8 | 6.6 | 8.8 |  | 4.0 |  | 0.059 | 0.74 | 0.92 |
| ΔIsc, carbachol (µA/cm²)^5^ | 42 | 25 | 19 |  | 54 | 15 | 36 |  | 20 |  | 0.68 | 0.35 | 0.80 |
| **Oxidative condition (monochloramine)** |  |  |  |  |  |  |  |  |  |  |  |  |  |
| Isc (µA/cm²) | 81 | 9 | 38 |  | 84 | 18 | 16 |  | 33 |  | 0.90 | 0.13 | 0.88 |
| TEER (Ω x cm²) | 34 | 65 | 49 |  | 42 | 56 | 38 |  | 8 |  | 0.57 | 0.058 | 0.43 |
| ΔIsc, glucose (µA/cm²) | 4.5 | 0.6 | 1.6 |  | 6.5 | 1.1 | 3.8 |  | 2.5 |  | 0.47 | 0.23 | 0.94 |
| ΔIsc, carbachol (µA/cm²) | 28 | 22 | 6 |  | 30 | 15 | 16 |  | 13 |  | 0.90 | 0.37 | 0.82 |

^1^Low numbers of available data due to frequent electrode saturation after tissue mounting in Ussing chambers.

^2^ Treat.: Treatment of sows pre- and post-partum (control *versus* antibiotic); age (d14 and d28, unweaned; d42 weaned from d28); treat.*age: treatment by age interaction.

^3^Isc: Short-circuit current.

^4^ TEER: Trans-epithelial electrical resistance.

^5^ΔIsc: Change in Isc induced by glucose or carbachol addition.
