# Supplementary material for: Maternal Antibiotic-Induced Early Changes in Microbial Colonization Selectively Modulate Colonic Permeability and Inducible Heat Shock Proteins, and Digesta Concentrations of Alkaline Phosphatase and TLR-Stimulants in Swine Offspring
Source: PLoS One. 2015 Feb 17;10(2):e0118092. doi: 10.1371/journal.pone.0118092 (PMC4331088; doi:10.1371/journal.pone.0118092)
Supplement: S3 Table — (DOCX) [file pone.0118092.s004.docx]

**Table S3. Alkaline phosphatase (AP) activity concentrations in colonic mucosa and in cecal and rectal digesta contents of pigs born to control or antibiotic-treated sows and slaughtered at different ages (LSmeans and SEM, n=10-12 per treatment).**

| *Sow’s treatment* | **Control** | | |  | **Antibiotic** | | |  |  |  | **Statistics (P =)^1^** | |  |
| --- | --- | --- | --- | --- | --- | --- | --- | --- | --- | --- | --- | --- | --- |
| *Offspring’s age* | **d14** | **d28** | **d42** |  | **d14** | **d28** | **d42** |  | **SEM** |  | **treat.** | **age** | **treat.*age** |
| **Colonic tissue** |  |  |  |  |  |  |  |  |  |  |  |  |  |
| µg AP/ mg protein | 1.12 | 1.50 | 1.82 |  | 1.88 | 2.16 | 1.96 |  | 0.45 |  | 0.18 | 0.55 | 0.76 |
| µg AP/ g mucosa | 51 | 69 | 77 |  | 90 | 98 | 71 |  | 22 |  | 0.29 | 0.79 | 0.56 |
| **Cecal digesta** |  |  |  |  |  |  |  |  |  |  |  |  |  |
| µg AP/ mg protein | 16.6 | 25.8 | 28.1 |  | 22.9 | 24.4 | 33.7 |  | 5.6 |  | 0.51 | 0.25 | 0.75 |
| µg AP/ g fresh digesta | 423 | 448 | 288 |  | 493 | 417 | 352 |  | 98 |  | 0.73 | 0.39 | 0.85 |
| **Rectal digesta** |  |  |  |  |  |  |  |  |  |  |  |  |  |
| µg AP/ mg protein | 9.4 | 11.1 | 7.2 |  | 15.6 | 7.3 | 2.6 |  | 2.8 |  | 0.73 | 0.066 | 0.12 |
| µg AP/ g fresh digesta | 296 | 267 | 104 |  | 500 | 227 | 115 |  | 60 |  | 0.26 | 0.04 | 0.11 |

^1^Treat.: Treatment of sows pre- and post-partum (control *versus* antibiotic); age (d14 and d28, unweaned, d42 weaned from d28); treat.*age: treatment by age interaction.
