# Supplementary material for: Maternal Antibiotic-Induced Early Changes in Microbial Colonization Selectively Modulate Colonic Permeability and Inducible Heat Shock Proteins, and Digesta Concentrations of Alkaline Phosphatase and TLR-Stimulants in Swine Offspring
Source: PLoS One. 2015 Feb 17;10(2):e0118092. doi: 10.1371/journal.pone.0118092 (PMC4331088; doi:10.1371/journal.pone.0118092)
Supplement: S4 Table — (DOCX) [file pone.0118092.s005.docx]

**Table S4. Crypt architecture of colonic mucosa in pigs born to control or antibiotic-treated sows and fed a low (LF) or a high (HF) fat diet between 140 and 169 days of age (LSmeans and SEM, n = 10 per treatment).**

| *Sow’s treatment* | **Control** | |  | **Antibiotic** | |  |  |  | **Statistics (P =)^1^** | |  |
| --- | --- | --- | --- | --- | --- | --- | --- | --- | --- | --- | --- |
| *Offspring’s diet* | **LF** | **HF** |  | **LF** | **HF** |  | **SEM** |  | **treat.** | **diet** | **treat.*diet** |
| Depth (µm) | 437 | 461 |  | 434 | 433 |  | 13 |  | 0.27 | 0.39 | 0.35 |
| Width (µm)^2^ | 69.0 | 68.0 |  | 64.3 | 66.7 |  | 1.5 |  | 0.067 | 0.64 | 0.26 |
| Perimeter (µm) | 994 | 1038 |  | 986 | 980 |  | 27 |  | 0.25 | 0.48 | 0.35 |
| Surface area (µm² x 10^3^) | 29.9 | 30.8 |  | 28.3 | 28.5 |  | 1.1 |  | 0.11 | 0.63 | 0.76 |

^1^Treat.: Treatment of sows pre- and post-partum (control *versus* antibiotic); diet (low *versus* high fat diet); treat.*diet: treatment by diet interaction

^2^Tendency for a treatment effect (P = 0.067): 68.5 (1.1) vs. 65.5 (1.1) µm for the pigs born to control and antibiotic-treated sows, respectively.
