# Supplementary material for: Maternal Antibiotic-Induced Early Changes in Microbial Colonization Selectively Modulate Colonic Permeability and Inducible Heat Shock Proteins, and Digesta Concentrations of Alkaline Phosphatase and TLR-Stimulants in Swine Offspring
Source: PLoS One. 2015 Feb 17;10(2):e0118092. doi: 10.1371/journal.pone.0118092 (PMC4331088; doi:10.1371/journal.pone.0118092)
Supplement: S5 Table — (DOCX) [file pone.0118092.s006.docx]

**Table S5. Electrophysiological characteristics of basal and monochloramine-stimulated colonic mucosa of pigs born to control or antibiotic-treated sows and fed a low (LF) or a high (HF) fat diet between 140 and 169 days of age (LSmeans and SEM, n = 8-10 per treatment).**

| *Sow’s treatment* | **Control** | |  | **Antibiotic** | |  |  |  | **Statistics (P =)^1^** | |  |
| --- | --- | --- | --- | --- | --- | --- | --- | --- | --- | --- | --- |
| *Offspring’s diet* | **LF** | **HF** |  | **LF** | **HF** |  | **SEM** |  | **treat.** | **diet** | **treat.*diet** |
| **Basal condition** |  |  |  |  |  |  |  |  |  |  |  |
| Isc (µA/cm²)^2^ | -7.8 | 0.0 |  | 12.9 | -35.5 |  | 24.1 |  | 0.80 | 0.25 | 0.25 |
| TEER (Ω x cm²)^3^ | 34.2 | 42.0 |  | 38.6 | 39.1 |  | 6.0 |  | 0.84 | 0.61 | 0.54 |
| ΔIsc, glucose (µA/cm²)^4^ | 0.6 | 6.7 |  | 0.5 | 2.6 |  | 3.8 |  | 0.47 | 0.24 | 0.68 |
| ΔIsc, carbachol (µA/cm²)^4^ | 58 | 58 |  | 56 | 29 |  | 28 |  | 0.60 | 0.53 | 0.63 |
| **Oxidative condition (monochloramine)** |  |  |  |  |  |  |  |  |  |  |  |
| Isc (µA/cm²) | 18.5 | 24.7 |  | 0.1 | 11.2 |  | 12.8 |  | 0.33 | 0.42 | 0.86 |
| TEER (Ω x cm²) | -5.9 | -0.1 |  | -3.0 | 3.2 |  | 4.4 |  | 0.73 | 0.19 | 0.97 |
| ΔIsc, glucose (µA/cm²) | -0.2 | -4.7 |  | 1.0 | 1.2 |  | 1.7 |  | 0.087 | 0.20 | 0.24 |
| ΔIsc, carbachol (µA/cm²) | 149 | 110 |  | 28 | 55 |  | 49 |  | 0.18 | 0.92 | 0.54 |

^1^Treat.: Treatment of sows pre- and post-partum (control *versus* antibiotic); diet (low *versus* high fat diet); treat.*diet: treatment by diet interaction.

²Isc: Short-circuit current.

^3^TEER: Trans-epithelial electrical resistance.

^4^Δ-Isc: Change in Isc induced by glucose or carbachol addition.
