# Supplementary material for: Maternal Antibiotic-Induced Early Changes in Microbial Colonization Selectively Modulate Colonic Permeability and Inducible Heat Shock Proteins, and Digesta Concentrations of Alkaline Phosphatase and TLR-Stimulants in Swine Offspring
Source: PLoS One. 2015 Feb 17;10(2):e0118092. doi: 10.1371/journal.pone.0118092 (PMC4331088; doi:10.1371/journal.pone.0118092)
Supplement: S6 Table — (DOCX) [file pone.0118092.s007.docx]

**Table S6. Concentrations of TLR-stimulants in rectal contents of pigs born to control or antibiotic-treated sows and fed a low (LF) or a high (HF) fat diet between 140 and 169 days of age (LSmeans and SEM, n = 8-10 per treatment).**

| *Sow’s treatment* | **Control** | |  | **Antibiotic** | |  |  |  | **Statistics (P =)^1^** | |  |
| --- | --- | --- | --- | --- | --- | --- | --- | --- | --- | --- | --- |
| *Offspring’s diet* | **LF** | **HF** |  | **LF** | **HF** |  | **SEM** |  | **treat.** | **diet** | **treat.*diet** |
| Rectal content dry matter (DM, %) | 24.1 | 23.9 |  | 23.1 | 21.8 |  | 1.15 |  | 0.21 | 0.48 | 0.63 |
| TLR2-Stimulant (µg/g DM) | 81 | 115 |  | 147 | 136 |  | 19 |  | 0.047 | 0.55 | 0.25 |
| TLR4-Stimulant (µg/g DM) | 7.6^b^ | 6.4^b^ |  | 8.5^b^ | 12.4^a^ |  | 1.5 |  | 0.036 | 0.34 | 0.082 |

^1^Treat.: Treatment of sows pre- and post-partum (control *versus* antibiotic); diet (low *versus* high fat diet); treat.*diet: treatment by diet interaction.

^a,b^ Means with different superscript letters in a row differ (P < 0.05).
